# How do trees respond to species mixing in experimental compared to observational studies?

**DOI:** 10.1002/ece3.5627

**Published:** 2019-09-10

**Authors:** Stephan Kambach, Eric Allan, Simon Bilodeau‐Gauthier, David A. Coomes, Josephine Haase, Tommaso Jucker, Georges Kunstler, Sandra Müller, Charles Nock, Alain Paquette, Fons van der Plas, Sophia Ratcliffe, Fabian Roger, Paloma Ruiz‐Benito, Michael Scherer‐Lorenzen, Harald Auge, Olivier Bouriaud, Bastien Castagneyrol, Jonas Dahlgren, Lars Gamfeldt, Hervé Jactel, Gerald Kändler, Julia Koricheva, Aleksi Lehtonen, Bart Muys, Quentin Ponette, Nuri Setiawan, Thomas Van de Peer, Kris Verheyen, Miguel A. Zavala, Helge Bruelheide

**Affiliations:** ^1^ Institute of Biology/Geobotany and Botanical Garden Martin Luther University Halle‐Wittenberg Halle Germany; ^2^ Department of Community Ecology Helmholtz‐Centre for Environmental Research - UFZ Halle Germany; ^3^ German Centre for Integrative Biodiversity Research (iDiv) Halle‐Jena‐Leipzig Leipzig Germany; ^4^ Institute of Plant Sciences University of Bern Bern Switzerland; ^5^ Centre for Development and Environment University of Bern Bern Switzerland; ^6^ Direction de la Recherche Forestière (DRF) Ministry of Forests, Wildlife and Parks Québec City QC Canada; ^7^ Forest Ecology and Conservation Group Department of Plant Sciences University of Cambridge Cambridge UK; ^8^ Geobotany Faculty of Biology University of Freiburg Freiburg Germany; ^9^ Department of Environmental Systems Science Institute for Terrestrial Ecosystems ETH Zurich Zurich Switzerland; ^10^ School of Biological Sciences University of Bristol Bristol UK; ^11^ Univ. Grenoble Alpes, Irstea, UR LESSEM Grenoble France; ^12^ Centre for Forest Research (CEF) Université du Québec à Montréal Montréal QC Canada; ^13^ Department of Systematic Botany and Functional Biodiversity Institute of Biology Leipzig University Leipzig Germany; ^14^ NBN Trust: Unit F Nottingham UK; ^15^ Centre for Environmental and Climate Research Lund University, Ekologihuset Lund Sweden; ^16^ Forest Ecology and Restoration Group Department of Life Sciences Universidad de Alcalá Alcalá de Henares Madrid Spain; ^17^ Department of Biology and Geology, Physics and Inorganic Chemistry Escuela Superior de Ciencias Experimentales y Tecnología Universidad Rey Juan Carlos Móstoles Madrid Spain; ^18^ University Stefan cel Mare of Suceava Suceava Romania; ^19^ Laboratory of Forest Inventory National Institute of Geographic and Forest Information (IGN) Nancy France; ^20^ BIOGECO INRA Université de Bordeaux Cestas France; ^21^ Swedish University of Agricultural Sciences Umeå Sweden; ^22^ Department of Marine Sciences University of Gothenburg Gothenburg Sweden; ^23^ Forest Research Institute Baden‐Wurttemberg Freiburg Germany; ^24^ School of Biological Sciences Royal Holloway University of London Egham UK; ^25^ Natural Resources Institute Finland (Luke) Helsinki Finland; ^26^ Department of Earth and Environmental Sciences University of Leuven Leuven Belgium; ^27^ Earth and Life Institute Environmental Sciences Université catholique de Louvain (UCLouvain) Louvain‐la‐Neuve Belgium; ^28^ Forest & Nature Lab Department of Environment Ghent University Gontrode Belgium

**Keywords:** biodiversity, ecosystem function and services, FunDivEUROPE, national forest inventories, productivity, species richness, synthesis, tree growth, TreeDivNet

## Abstract

For decades, ecologists have investigated the effects of tree species diversity on tree productivity at different scales and with different approaches ranging from observational to experimental study designs. Using data from five European national forest inventories (16,773 plots), six tree species diversity experiments (584 plots), and six networks of comparative plots (169 plots), we tested whether tree species growth responses to species mixing are consistent and therefore transferrable between those different research approaches. Our results confirm the general positive effect of tree species mixing on species growth (16% on average) but we found no consistency in species‐specific responses to mixing between any of the three approaches, even after restricting comparisons to only those plots that shared similar mixtures compositions and forest types. These findings highlight the necessity to consider results from different research approaches when selecting species mixtures that should maximize positive forest biodiversity and functioning relationships.

## Graphical abstract

Using data from five European national forest inventories, six tree species diversity experiments, and six networks of comparative plots, we confirmed a general positive of species mixing on tree growth. Species‐specific responses to mixing were inconsistent between any of the tree approaches, even after restricting comparisons to only those plots that shared similar mixtures compositions and forest types. We thereby highlight the necessity to consider results from different research approaches to select species mixtures for maximizing biodiversity effects on tree growth.

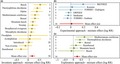

## 1 INTRODUCTION

The provisioning of ecosystem services beneficial to human well‐being strongly relies on plant diversity (Cardinale et al., 2012). Decreases in primary producer diversity can impact ecosystem functioning and decrease ecosystem productivity and stability (Cardinale et al., 2012; Hooper et al., 2012), a phenomenon especially well studied in grassland ecosystems (e.g., Isbell et al., 2015; Reich et al., 2012; Tilman et al., 1997) where log species richness and log productivity are often linearly related (Craven et al., 2016; Hector et al., 1999; Tilman et al., 1997). In forest ecosystems, systematic research on the effects of species mixing on wood production dates back to the foundations of modern forestry (Hartig, 1791). Current global synthesis studies concluded that, across the different forest biomes, a positive relationship between tree diversity and stand productivity prevails (Liang et al., 2016; Scherer‐Lorenzen, 2014; Zhang, Chen, & Reich, 2012).

The relationship between tree diversity and productivity has already been studied using different research approaches (Table 1), starting with the analysis of forest inventories (Hartig, 1791; Schwappach, 1912; Wiedemann, 1943), followed by silvicultural trials and tree diversity experiments (Bruelheide et al., 2014; Koricheva, 2002; Pretzsch, 2005; Scherer‐Lorenzen et al., 2005; Tobner, Paquette, Reich, Gravel, & Messier, 2014; Verheyen et al., 2016) and more recently by the selection of comparative plots in mature forests (Baeten et al., 2013; Bruelheide et al., 2011; Fischer et al., 2010). Forest inventories usually cover large numbers of uniformly distributed plots across multiple forest types and large environmental gradients. Tree diversity experiments, in contrast, consist of spatially restricted, replicated plantations of different tree species compositions and levels of tree species diversity and have minimal variation in environmental conditions. Comparative study plots (Bruelheide et al., 2011) or “exploratories” (Fischer et al., 2010) consist of survey plots within mature forests selected to contain replicated levels of tree species diversity and compositions while at the same time controlling for differences in community structure and environmental conditions. They can thus be regarded as an intermediate approach that combines aspects of forest inventories and tree diversity experiments.

**Table 1:** Summary of the advantages, disadvantages, and exemplary findings on the relationship between tree species diversity and tree growth or stand‐level biomass production in three different research approaches. Figures depict the characteristics of the research approaches: **r**epresentativeness (i.e., the anticipated transferability of the findings to existing forests), **c**omprehensiveness (i.e., the number of ecosystem functions and properties that can be feasibly quantified), and **o**rthogonality (i.e., the ability to quantify the effect of tree diversity against a background of variation); Figures are based on Nadrowski et al. (2010) and Jucker et al. (2016) and published on http://project.fundiveurope.eu

Research approach	Advantages	Disadvantages	Reported effects of tree diversity on productivity
Tree diversity experiments 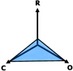	Solid statistical design Can include species mixtures that do not occur naturally Minimal variation in environmental characteristics Diversity orthogonal to other drivers of function Causal inference possible	Fixed number of tree species and combinations Cover only limited environmental gradients	Global network of tree diversity experiments (Verheyen et al., 2016), http://www.treedivnet.ugent.be
			Positive (Pretzsch, 2005; Fichtner et al., 2017; Erskine, Lamb, & Bristow, 2006; Potvin & Gotelli, 2008; Haase et al., 2015)
			Nonsignificant (Tobner et al., 2016; Nguyen, Herbohn, Firn, & Lamb, 2012; Guo & Ren, 2014)
			Negative (Firn, Erskine, & Lamb, 2007)
Comparative forest plots (exploratories) 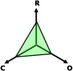	Controlled species composition Intermediate variation in stand characteristics Diversity as orthogonal as possible to other drivers of function Intermediate gradient in environmental conditions Can be established in mature forests	Limited number of tree species Causal inference is difficult	Positive (Barrufol et al., 2013; Jucker, Bouriaud, Avacaritei, & Coomes, 2014)
			Negative (Jacob, Leuschner, & Thomas, 2010)
Forest inventories 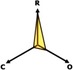	Large number of plots Vast geographic extend Large gradients in ‐ Species compositions ‐ Stand characteristics ‐ Environmental conditions Highly representative	Large heterogeneity can confound diversity signals Design originally not developed to study biodiversity‐ecosystem function relationships Causal inference not possible	Positive (Liang et al., 2016; Paquette & Messier, 2011; Vilà et al., 2013; Ruiz‐Benito et al., 2014, 2017; Ratcliffe et al., 2017; Madrigal‐González et al., 2016; Guo & Ren, 2014; Vilà et al., 2007)
			Nonsignificant (Szwagrzyk & Gazda, 2007; Moser & Hansen, 2009; Long & Shaw, 2010; Vayreda, Gracia, Canadell, & Retana, 2012)
			Hump‐shaped (Gamfeldt et al., 2013)
			Negative (Mina, Huber, Forrester, Thürig, & Rohner, 2017)

Regardless of the approach applied, most previous research on forest diversity‐productivity relationships focussed on the effects of tree species diversity on the productivity of the community (e.g., Homeier, Breckle, Günter, Rollenbeck, & Leuschner, 2010; Jucker et al., 2016; Liang et al., 2016; Paquette & Messier, 2011; Ruiz‐Benito et al., 2014; Vilà et al., 2013). In theory, any positive effect of species diversity could stem from either positive interactions between the co‐occurring species (complementarity effects, Loreau & Hector, 2001) or from the admixing of one or few exceptionally productive or dominating species (selection effects, Loreau & Hector, 2001). Depending on the forest ecosystem, species‐specific growth responses to increasing tree diversity can be consistently positive (Chamagne et al., 2017; Liang et al., 2016) or variable, depending on the species and context (Baeten et al., 2019; Jucker, Bouriaud, Avacaritei, Dănilă, et al., 2014; Ratcliffe, Holzwarth, Nadrowski, Levick, & Wirth, 2015; del Río et al., 2017; Tobner et al., 2016). It is unclear to what extent these differences in species responses to tree diversity are caused by differences in species‐specific characteristics (Fichtner et al., 2017; Williams, Paquette, Cavender‐Bares, Messier, & Reich, 2017) or differences in study design. Comparing species‐specific responses to mixing between the different research approaches could help to determine which species generally benefit, suffer, or show divergent responses to increases in tree species diversity. Restricting these comparisons to only the set of tree species and forest types that are shared between research approaches should furthermore reduce the confounding effects of species compositions and large scale environmental context‐dependency and leave mainly the effects of local environmental context‐dependency and differences in stand structure.

In the FunDivEUROPE research network (functional significance of forest diversity in Europe, Baeten et al., 2013), all three previously described approaches (experiments, exploratories and inventories) were applied throughout Europe to study the effects of tree diversity on forest ecosystem functioning. The three approaches partly overlap in their species pools, although there are differences in species compositions as well as successional, structural, climatic and edaphic plot conditions. Syntheses across all three approaches can thus be applied to test whether most tree species respond consistently to species mixing. Identifying tree species that display consistent responses between different approaches and different forest types would furthermore allow the isolation of general patterns from context‐dependent effects.

With this study, we provide a first comparison of the growth response of a large set of tree species to species mixing across three distinct research approaches (tree diversity experiments, networks of comparative plots and forest inventories). We tested the following hypotheses: (H1) across all species and research approaches, tree species growth is higher in mixed than in monospecific tree communities, (H2) across all species and research approaches, the effect of tree species mixing on species growth linearly increases with the logarithm of the number of admixed tree species (two, three or higher species mixtures), and (H3) species' aggregated responses to mixing are correlated between different research approaches. We furthermore hypothesized that species' responses to mixing will become more consistent between the three research approaches, if we compare only matching species compositions. (H4). The findings of this study should deepen our understanding of the species, environmental conditions, and research designs for which consistent positive diversity‐ecosystem functioning relationships can be expected.

## 2 METHODS

**Figure 1:**
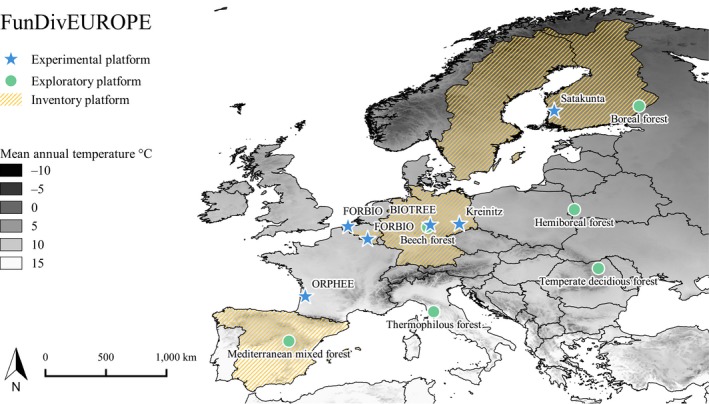
Location of the research approaches compiled in this study. Shaded countries: national forest inventories (16,773 plots), stars: tree diversity experiments (584 plots), and black dots: forest exploratories (169 plots)

Within the framework of the European FunDivEUROPE project (http://www.fundiveurope.eu), the significance of forest biodiversity for ecosystem functioning across Europe was investigated with three complementary research approaches (tree diversity experiments, networks of comparative plots in established forests, and forest inventories). All approaches share a similar subset of tree species and forest types and were established in regions with similar climatic conditions (see Appendices S1–S4 and Baeten et al., 2013). The approaches differed in how well they represented existing mature forests, the comprehensiveness of the studied tree species and environmental gradients and the extent to which potentially confounding effects could mask the effects of tree species diversity (“orthogonality”, see Table 1, Figure 1 and Nadrowski, Wirth, & Scherer‐Lorenzen, 2010).

### 2.1 Research approaches

The experimental research approach contained growth measurements from six European tree diversity experiments, which individually covered species richness gradients from one up to six tree species, with different mixtures replicated at each level of species richness. Detailed information on the design and tree species composition of each diversity experiment is reported in Appendix S1 and on http://www.treedivnet.ugent.be. Tree sizes were measured in 2014 and reported as either tree's diameter at breast height and the derived basal area (in 114 plots of the Satakunta, 96 plots of the Kreinitz and 32 plots of the BIOTREE experiment), tree height (in 256 plots of the ORPHEE experiment), or diameter at ground height (in 42 plots of the FORBIO—Zedelgem and 44 plots of the FORBIO—Gedinne experiment).

The exploratory research approach contained a network of 209 comparative study plots that were established in six different European forest types. In each forest type, between three and five regionally common, and from a forestry perspective, important tree species were selected as target species. Plots representing species richness gradients from one up to five target tree species were established in 2011. Similar to the experimental approach, different compositions per tree species richness level were chosen to ensure that diversity effects were not confounded with the effects of diluting individual species in plots of higher species richness and the plots were selected to minimize any covariation between environmental conditions (e.g., geology, soil texture and depth and topography) and tree species richness and composition. The study design as well as the forest characteristics and tree species compositions are described in Appendices S1–S4 and in Baeten et al. (2013). Within each plot, all trees with a dbh of more than 7.5 cm were mapped and identified. From a subset of trees, wood core samples were taken and, based on radial stem increments between 1999 and 2010, the mean annual increase in basal area per tree was calculated (m^2^ ha^−1^ year^−1^, Appendix S7, see Jucker, Bouriaud, Avacaritei, & Coomes, 2014). The number of plots per forest type was as follows: beech forest (24), boreal forest (28), hemiboreal forest (25), Mediterranean coniferous forest (33), mountainous beech forest (26), and thermophilous deciduous forest (33). We calculated for each plot, the proportion that was covered by each tree species and classified each plot as either a monospecific, two, three or higher species mixture, where the most dominant species must cover more than 90% and none of the “nondominant” species more than 10% of a plot's summed basal area.

The inventory research approach contained harmonized forest plots from five national forest inventories (Finland, Sweden, Germany, Belgium—Wallonia, and Spain) that had been surveyed at least twice. Details can be found in Appendix S5 and in Ratcliffe et al. (2016). In short, for all trees with a dbh of 10 cm or more, we extracted the tree status (ingrowth, survivor, dead due to natural mortality or harvesting) and basal area (expressed as m^2^/ha) from the two most recent survey dates. We discarded all plots with indications of harvesting activities between survey dates. Tree species names were harmonized following the Atlas Florae Europaeae (Kurtto, Sennikov, & Lampinen, 2013). Within each plot, we calculated the proportion of total basal area that was belonged to each tree species. Analogous to the exploratory approach, we classified each plot as either a monospecific, two, three or higher species mixture. After discarding all plots that did not meet these criteria, we retained 47,754 plots in the inventory dataset (see Appendix S4 for a more detailed description of the classification criteria).

### 2.2 Environmental data

For each plot of the three research approaches, we extracted mean annual temperature, temperature seasonality (standard deviation of mean monthly temperatures), annual precipitation, and precipitation seasonality (standard deviation of mean monthly precipitation) from the WorldClim dataset (interpolated from measurements taken between 1960 and to 1990 and at a spatial resolution of one square kilometer, Hijmans, Cameron, Parra, Jones, & Jarvis, 2005) and the slope from the GTOPO30—digital elevation model with a spatial resolution of one square kilometer (data available from the U.S. Geological Survey).

### 2.3 Data preparation

For each plot of the experimental, exploratory and inventory approach, we calculated for every target/dominant species the yearly summed increase in basal area, dbh, tree height, or diameter at ground height (based on the respective growth measurement). These summed growth estimates were divided by the number of trees in the experiments and by the summed basal area (m^2^ ha^‐1^) of the respective tree species in the exploratory and inventory approach to obtain growth estimates (hereafter “species growth”) that are not biased by potentially uneven species proportions.

Within each forest type and tree diversity experiment, we quantified the effect of species mixing on species growth as the mean log response ratio, defined as species growth in mixed divided by species growth in monospecific plots of comparable stand conditions (i.e., within the same dataset and forest type). In the exploratory approach, no monospecific plots of *Acer pseudoplatanus* L. were found in the beech forest and no monospecific plots of *Betula spec*. and *Quercus robur* L. were found in the hemiboreal forest. For these three species, we could not calculate the effect sizes in the respective forest types which, thus, reduced our exploratory dataset to 169 plots.

In the inventory approach, mixed and monospecific plots within the same forest type could differ considerably in stand conditions (e.g., in climate, tree community structure, and edaphic conditions). To partly control for these potentially confounding differences, we first assigned pairs of monospecific and mixed plots that were most similar regarding stand and environmental conditions and subsequently calculated the effect size for each pair of plots. The dissimilarity in stand and environmental conditions was quantified as the Euclidean distance in normalized plot‐level values (i.e., subtracted by the mean and divided by the standard deviation) of mean annual temperature, temperature seasonality, annual precipitation, precipitation seasonality, slope and the sum and coefficient of variation of trees' basal area (m^2^/ha). The latter two were included in order to account for potential effects of stand age and evenness (e.g., Zhang, Chen, & Reich, 2012). The pairs of most similar mixed and monospecific plots (i.e., with the smallest Euclidean distances) were selected via a nearest neighbor matching algorithm (Ho, Imai, King, & Stuart, 2007; Ho, Imai, King, & Stuart, 2011) that minimized, within each forest type, the summed Euclidean distances. This was done for each species separately, to compare species growth in mixed versus monospecific plots. A three‐species mixture could thus be paired with up to three monospecific plots of its component species (note that a monospecific plot could only be assigned to one mixture plot). To eliminate comparisons between very different stand conditions, we discarded all plot pairs with distance values that were above the 90% percentile of all distances (Figure S9). The locations of the remaining 16,773 plots are shown in Figure S6. All plots were assigned to one of the following forest types, listed in the EEA Technical Report 9 (Barbati, Corona, & Marchetti, 2007): acidophilous oak and oak‐birch forest (104 plots), alpine coniferous forest (615), beech forest (475), boreal forest (2,440), broadleaved evergreen forest (2,129), floodplain forest (20), hemiboreal forest and nemoral coniferous and mixed broadleaved‐coniferous forest (1,391), plantations and exotic forest (1,088), Mediterranean coniferous forest (6,098), mesophytic deciduous forest (582), mountain beech forest (426), nonriverine alder, birch or aspen forest (254), mire and swamp forest (204) or thermophilous deciduous forest (947). Because the survey dates and the methods applied to measure tree growth differed between the different national forest inventories, we noted the country of each mixed and monospecific plot to later statistically account for it.

In order to narrow down the comparisons of mixing effects to only those tree species and community compositions that were shared between the three approaches, we created three data subsets that included only those species and mixtures that were present in two datasets, that is, (a) the experimental and exploratory, (b) the experimental and inventory, and (c) the exploratory and inventory approach (Table S4).

### 2.4 Statistical analysis

Separately for each tree diversity experiment and each forest type within the exploratory or the inventory dataset, we calculated for every tree species the separate mean log response ratio (hereafter “effect size”) of the species' growth in either all 2, 3 or higher species mixtures divided by the growth in the respective monospecific plots of that forest type/diversity experiment. The whole data preparation procedure up to the point of the calculation of effect sizes is briefly summarized in Appendix S8.

We tested hypothesis H1 (i.e., a general positive effect of tree species mixing on species growth) by testing for significance of the grand mean effect size (i.e., the intercept) with a linear random‐effects model. The model included effect sizes as the dependent variable and the identity of the experiment/forest type and, in the case of the inventory approach, the countries of the compared plots, as random effects. In the national forest inventory dataset, certain species could have multiple effect sizes within the same forest type and species richness level (because we did not pool effect sizes between different countries). Those multiple effect sizes were assigned an accordingly lower weight in the following linear model (calculated as one divided by the number of multiple effect sizes). The resulting grand mean effect size was deemed significant, if the approximated 95% confidence interval (intercept ± 1.96 × *SE*) did not include zero. We tested the differences between approaches by including the research approach as a categorical predictor variable in the mixed‐effects model.

Hypothesis H2 (i.e., a positive effect of log species richness on the species' mean log response ratios) was tested with linear mixed‐effects models that included the effect sizes as the dependent variable, log species richness as the predictor variable and the identity of the forest type or experiment and, in case of the inventory approach, the countries of the compared plots as a nested random effect. In contrast to the model applied to test H1, we assigned equal weights to all effect sizes. In the inventory approach, we weighted effects sizes by the inverse of the number of effect sizes for the same species in the same forest type (this number could vary when plots from different forest inventories were assigned to the same forest type).

H2 was then tested by comparing the variance explained with the full model versus the variance explained with solely the random effects (analysis of variance).

In order to test hypothesis H3 (i.e., the consistency in species‐specific responses to mixing across the research approaches), we fitted separate mixed‐effects models per approach (for the experimental, exploratory, and inventory approach, respectively). These models included the identity of the tree species as a predictor variable and the random‐effects structure was adapted from the model that was applied to test H1. The intercept of each model was set to zero. From each model, we then extracted the coefficient estimates for the respective tree species included. The consistency in species responses was then assessed by testing the significance of the rank‐based correlation coefficients (Kendall's tau) between the coefficient estimates of species that were shared between different approaches (separately for the experiments‐exploratories, experiments‐inventories, and exploratories‐inventories comparison).

Hypothesis H4 (i.e., the proposed increase in the consistency of species responses to mixing when the comparisons of approaches were restricted to only those community compositions and forest types that are shared between the approaches) was tested analogous to H3, but this time based on datasets restricted to tree species occurring in the same compositions and forest types in the compared research approaches (listed in Table S4). The obtained Kendall's tau values were then compared to the tau values that were obtained from the unrestricted datasets.

All analyses were conducted in *R* (R Core Team, 2018) using the following packages: *ggplot2* for graphical representations (Wickham, 2009), *cluster* for distance matrix calculations (Maechler, Rousseeuw, Struyf, Hubert, & Hornik, 2015, p. 20), *MatchIt* for finding pairs of similar mixed and monospecific plots (Ho et al., 2011)*, lme4* for calculating linear random‐ and mixed‐effects models (Bates, Mächler, Bolker, & Walker, 2015), and *raster* for extracting the *WorldClim* data (Hijmans, 2013).

## 3 RESULTS

**Figure 2:**
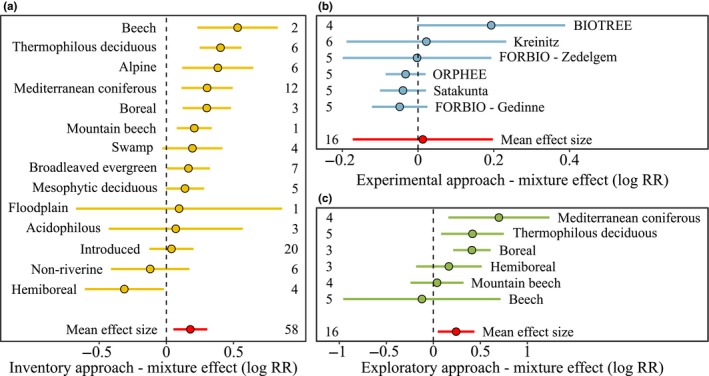
Mean effect sizes (log response ratios) of tree species growth in mixed compared to monospecific plots averaged per forest type/tree diversity experiment in the three different research approaches: (a) forest inventories, (b) tree diversity experiments, and (c) forest exploratories. Numbers denote the number of tree species for which effect sizes could be calculated. Different forest types/diversity experiment could overlap in the analyzed tree species. Thus, the species of the grand mean effect sizes are lower than the summed species numbers

**Figure 3:**
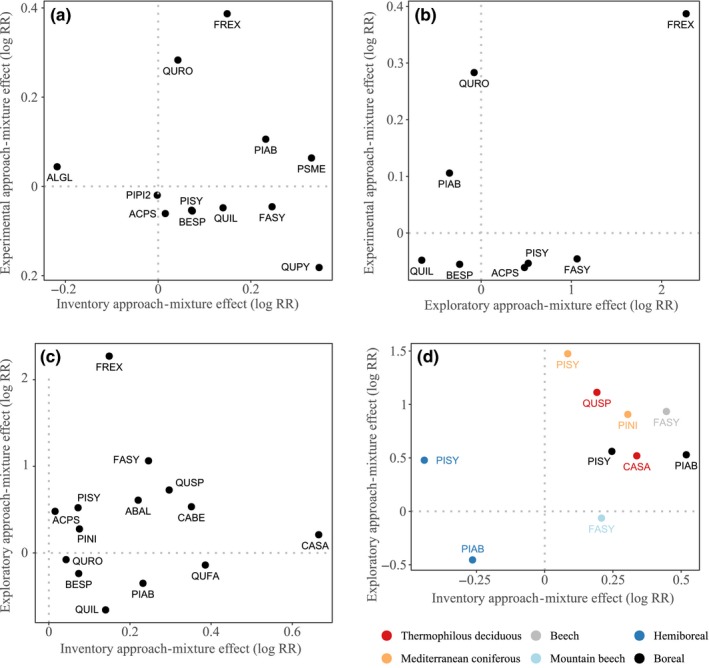
Comparison of tree species mean effect sizes (log response ratios) of growth in mixed compared to monospecific plots obtained from three different research approaches (experimental, exploratory, and inventory approach). Depicted are the mean effect sizes of only those species that were shared between the compared research approaches (a: experiments vs. inventories, b: experiments vs. exploratories, c: exploratories vs. inventories, and d: exploratories vs. inventories when species responses were separated by forest type). Abbreviations: ABAL: *Abies alba* Mill., ACPS: *Acer pseudoplatanus* L., BESP: *Betula spec*., ALGL: *Alnus glutinosa* (L.) Gaertn., CABE: *Carpinus betulus* L., CASA: *Castanea sativa* Mill., FASY: *Fagus sylvatica* L., FREX: *Fraxinus excelsior* L., PIAB: *Picea abies* (L.) H.Karst., PINI: *Pinus nigra* J.F.Arnold, PIPI2: *Pinus pinea* L., PISY: *Pinus sylvestris* L., PSME: *Pseudotsuga menziesii* (Mirb.) Franco, QUFA: *Quercus faginea* Lam., QUIL: *Quercus ilex* L., QUPY: *Quercus pyrenaica* Willd., QURO: *Quercus robur* L., QUSP: *Quercus spec* – combines *Q. petraea* and *Q. pubescens Willd. (Q. humilis)* (Table S2)

(H1) When calculated across all three research approaches (experiments, exploratories, and inventories), the grand mean effect size of species mixing (i.e., the average log response ratio of species growth in mixed compared to monospecific plots) was significantly positive (approximated 95% confidence interval: 0.05–0.25). On average, species showed 16% higher growth in mixed compared to monospecific plots. When calculated separately for each research approach, both the inventory and exploratory dataset yielded significantly positive mean effect sizes (on average, species growth was 27% and 20% higher in mixed compared to monospecific plots of the exploratory and inventory approach, respectively, Figure 2), whereas the mean effect size of the experimental approach was nonsignificant (on average, species growth was 1% higher in mixed compared to monospecific plots, Figure 2). In the experimental approach, none of the mean effect sizes (average species log response ratios) of the individual diversity experiments was significantly different from zero. In the exploratory approach, significantly positive mean effect sizes were found in Mediterranean coniferous, thermophilous deciduous, and boreal forests. In the inventory approach, significantly positive mean effect sizes were found in beech, thermophilous deciduous, alpine, Mediterranean coniferous, boreal, and mountain beech forests.

(H2) Including log tree species richness as a predictor variable did not explain a significant amount of variation in species' effect sizes (*F*_df:1,299.75_ = 0.99, *p* = .32).

(H3) Between the different research approaches, tree species responses to mixing (i.e., the model coefficient estimates) were highly inconsistent (Figure 3). All Kendall's tau values ranked between 0.55 and 0.94 and were nonsignificant (*p*‐values ranged from .55 to .94). *Fraxinus excelsior* L. was the only species to exhibit consistent, and positive, effects sizes in all three research approaches (Figure 3).

(H4) Restricting the comparisons to only those species compositions and forest types that were shared between the compared research approaches did not lead to stronger correlations between species' coefficient estimates of different approaches (Figure S10). Kendall's tau values ranged from −0.2 to −0.06 and the respective *p*‐values ranged from .72 to .84.

## 4 DISCUSSION

In this study, we compiled tree growth data from three European research initiatives that used different research approaches (tree diversity experiments, networks of comparative “exploratory” plots in established forests, and national forest inventories) to summarize the effects of tree species mixing on the growth of 64 tree species.

Based on this extensive dataset, we conducted, to our knowledge, the first study on the transferability of the response of tree species growth to mixing from experiments to forest exploratories and national forest inventories. Our results confirmed our hypothesis of a general positive effect of tree species mixing on species growth across the three research approaches, although this effect was nonsignificant in the experiments. This finding is in accordance with the meta‐analysis of Piotto (2008) who also found that tree species generally exhibit higher growth in mixed compared to monospecific communities. In the exploratory and inventory dataset, tree species showed, on average, an increase of 27% and 20% in growth in mixed as compared to monospecific stands. Studies that investigated the effect of species mixing on the productivity of the whole tree community (as opposed to the growth of the individual species) reported positive effects of comparable magnitude. Tree communities exhibited a 21% higher productivity in mixed, as compared to their respective monocultures in the Spanish forest inventory (Ruiz‐Benito et al., 2014) and 24% higher productivity across the national forest inventories of France, the Netherlands, Spain, Sweden, and Switzerland (Vilà et al., 2013).

Previous analyses of the published literature (Zhang, Chen, & Reich, 2012), the Spanish national forest inventory (Ruiz‐Benito et al., 2014), and a global forest dataset (Liang et al., 2016) all found that the productivity of the whole tree community increases with the number of mixed tree species. In our analyses of individual species, however, we could not find such an increase in the magnitude of the mixing effect with the number of admixed tree species.

Regarding the exploratory approach, our results confirmed the findings of Jucker, Bouriaud, Avacaritei, and Coomes (2014), who previously analyzed the same exploratory dataset, and also found positive effects of species mixing on plot productivity in the Mediterranean coniferous, thermophilous deciduous and boreal forests type. Our findings are also in line with studies that investigated the same inventory dataset and found positive effect of tree diversity on the productivity of the whole tree community (Ruiz‐Benito et al., 2014; Ratcliffe et al., 2016; Ruiz‐Benito et al., 2017), although we investigated the effects on individual species and manipulated the inventory dataset to make it compatible to the exploratories.

Our results further suggested that species mixing mostly benefitted those species that grew in forest types with relatively cold (boreal and alpine forests) or hot climates (Mediterranean coniferous and thermophilous deciduous forests). These observations are in line with an analysis of an eastern Canadian forest inventory dataset that likewise found stronger positive effects of tree diversity on stand productivity in boreal as compared in temperate forests (Paquette & Messier, 2011). Together, these findings broadly support the stress‐gradient hypothesis, stating that positive interactions prevail in more stressful conditions (e.g., cold or dry), resulting in higher relative diversity effects than in more benign conditions (Forrester & Bauhus, 2016). We found consistent species responses to mixing between the exploratory and inventory approach only for those three forest types with the most stressful climatic conditions. However, for the remaining three forest types that were shared between both approaches and found in intermediate conditions, we found no consistency in the significance or even direction of the mixing effect. This limited transferability of mixing effects between approaches, already indicated that scaling of diversity effects across approaches might problematic.

Consequently, we found that species‐specific responses to mixing were largely inconsistent between all three approaches, even after restricting the datasets to plots of only those species compositions and forest types that were shared between the different approaches. These observed inconsistencies likely resulted from unaccounted but influential drivers of forest diversity and functioning relationships, like tree density, size heterogeneity, and successional status (Lasky et al., 2014).

In accordance with a recent global meta‐analysis (Duffy, Godwin, & Cardinale, 2017), we found tree diversity effects on productivity to be generally stronger in natural as compared to experimental study designs. We must point out that the tree diversity experiments included in this study were not planted to represent mature forests, but to isolate the effects of tree species richness and functional diversity on ecosystem functioning. Since those experimental forests were still in juvenile phases they usually lacked successional trajectories that lead to the replacement of underperforming species. Tree diversity experiments might therefore still harbor maladapted species that could not compete in mature forests with a similar climate. In the inventory dataset, however, trees were usually planted and managed to maximize wood production and financial return. We tried to minimize, but could not rule out the effects of local plot conditions on tree productivity. A number of plots might display both, a higher productivity and a higher tree species richness, simply because of the prevailing favorable climatic and edaphic conditions.

Differences in the climatic conditions can generally lead to different forest biodiversity‐productivity relationships (Paquette & Messier, 2011; Jucker et al., 2016; Ratcliffe et al., 2017). Although the three compared research approaches were established in overlapping climatic conditions they still varied in climatic and probably also edaphic conditions. Madrigal‐González et al. (2016) furthermore demonstrated that the impact of the diversity of neighboring trees on tree growth can be mediated by an interaction between tree size and climatic conditions. More specifically, across the national forest inventories of Finland, Germany, Spain, Sweden and Belgium‐Wallonia, Madrigal‐González et al. (2016) found that smaller trees benefitted from a complementary (i.e., functionally divergent) neighborhood only in the coldest and intermediate regions whereas larger trees benefitted from complementarity only in the warmest regions. With the approach applied in this study (i.e., the comparison of mean species growth between mixed and monospecific plots), we could not account for the potentially confounding differences in tree sizes and especially the interaction with prevailing climatic conditions.

Herbivore pressure is another factor that likely varied between the three approaches. Except for the Satakunta site, all tree diversity experiments were fenced to exclude game species and safeguard the successful establishment of all planted trees. In the inventory, and even more in the exploratory approach, the juvenile trees are exposed to pressure by game species, which are known to be affected by tree species richness (Milligan & Koricheva, 2013; Ohse, Seele, Holzwarth, & Wirth, 2017).

The effects of tree diversity on forest functioning are scale‐dependent, meaning that significance can change with the size of the surveyed forest plots (Wang et al., 2016). Inconsistencies in species‐specific responses could thus partly result from differences plot size and spatial extent between the compared research approaches.

In summary, all of the proposed factors might have contributed to the inconsistency of species‐specific responses to mixing between tree diversity experiments and established forests. On the one hand, these results impede clear recommendations for forest owners on how to jointly maximize forest diversity and productivity. On the other hand, our results unequivocally demonstrated that not even one of the 64 investigated tree species generally suffers from species mixing. Beside the hemiboreal forests in the inventory approach, most tree species were, on average, either not significantly or even positively affected by species mixing. We thus concluded that many, if not most, monospecific stands can be diversified without negative or with positive effects on wood production.

Future research will be needed to answer (a) what are underlying causes that lead to different diversity‐functioning relationships between observational and experimental research approaches and (b) what are the species‐specific abiotic and biotic requirements that maximize the productivity in mixed and monospecific communities. These findings will be essential to devise forest management practices that can maximize synergies between wood production and the safeguarding of forest diversity in Europe (Chamagne et al., 2017).

## CONFLICT OF INTEREST

None declared.

## AUTHOR'S CONTRIBUTIONS

M.S.‐L., K.V., J.K., H.J., H.B., H.A., D.A.C., B.M., and Q.P. designed the FunDivEUROPE experimental and exploratory platforms. S.R., P.R.‐B., A.L., G.K., J.D., and M.A.Z. contributed to the harmonization of the NFI datasets. The original ideas of this study were conceived by E.A., L.G., M.S.‐L., F.v.d.P., H.B., O.B., S.B.‐G., J.H., T.J., G.K., S.K., S.M., C.N., A.P., Q.P., F.R., S.R., and P. R.‐B. during an sDiv workshop that was organized by E.A. and L.G. The data were collated by O.B., D.A.C., J.D., T.J., J.H., J.K., Q.P., and T.V.d.P. S.K. performed the statistical analyses with the support of H.B., J.H., G.K., F.v.d.P., S.R., and P.R.‐B. S.K. wrote the first draft of the manuscript to which all co‐authors contributed critically and gave the final approval for publication.

## Data Availability

Information on the availability of the National Forest Inventory datasets can be found on the following websites: Finnland – http://www.metla.fi/ohjelma/vmi/info-en.htm, Germany – http://bwi.info/?xml:lang=en, Spain – http://www.magrama.gob.es/es/desarrollo-rural/temas/politica-forestal/inventario-cartografia/inventario-forestal-nacional, Sweden – http://www.slu.se/nfi – http://iprfw.spw.wallonie.be. Data‐requests regarding the tree diversity experiments should be sent to the respective data holders/contact persons listed at http://www.treedivnet.ugent.be. Data‐requests regarding the comparative forest plots should be sent to the respective data holder listed at http://fundiv.befdata.biow.uni-leipzig.de.
