# Supplementary material for: How do trees respond to species mixing in experimental compared to observational studies?
Source: Ecol Evol. 2019 Sep 10;9(19):11254–65. doi: 10.1002/ece3.5627 (PMC6802375; doi:10.1002/ece3.5627)

**Table S1.1.** Characteristics of national forest inventories

| **National forest inventory** | **Number of plots** | **Survey dates** | **Sample plot design** | **Plot size (m²)** |
| --- | --- | --- | --- | --- |
| Finland | 1,066 | Subset of NFI 8:  1985-1986 and 1995 | Cluster design, number and grid size depend on location, see text for details | 100, 300 |
| Sweden | 3,863 | Inventories 2005-2007 and 2008-2010 | Cluster design, number and grid size depend on region. Tract size is between 300-1800 m in length. | 38, 314 |
| Germany | 7,887 | BWI 1 (1986-1990) and BWI 2 (2001-2002) | Cluster design, 4 subplots. Grid size depends on region. Standard grid size is 4 by 4 km | BAF 4 m^2^ ha^-1^ |
| Wallonia | 48 | Inventories 1994-2003 and 2008-2011 | 1km by 0.5 km grid of single sample plots | 63, 254, 1017 |
| Spain | 14,883 | SFI 2 (1986-1996) and SFI 3 (1997-2007) | 1 km by 1 km grid of single sample plots | 79, 315, 707, 1964 |

**Table S1.2.** Characteristics of tree diversity experiments

| **Tree diversity experiment** | **Country** | **Contact** | **Set-up** | **Growth measurement** | **Species richness** | **Tree species** |
| --- | --- | --- | --- | --- | --- | --- |
| Satakunta | Finland | www.sataforestdiversity.org | 114 plots in 3 sites with a density of 169 trees per 400 m², planted in 1999 and partially thinned in 2013 | Diameter at breast height | 1, 2, 3, 5 | ALGL, BESP, LASI, PIAB, PISY |
| Kreinitz | Germany | www.treedivnet.ugent.  be/ExpKreinitz.html | 96 plots in 1 site with a density of 36 trees per 25 m² plot, planted in 2000 | Basal area | 1, 2, 3, 5, 6 | FASY, FREX, PIAB, PISY, QUSP, TICO |
| BIOTREE - Kaltenborn | Germany | www.biotree.uni-freiburg.de/deutsch/index.html | 16 plots (each divided in 2 subplots) in 1 site with a mean density of 1944 trees per 0.6 ha plot, planted in 2004 and partially thinned | Basal area | 1, 2, 3, 4 | FASY, PIAB, PSME, QURO |
| FORBIO - Gedinne | Belgium | www.treedivbelgium.  ugent.be | 44 plots in 2 sites with a density of 757 trees per average plot of 1670 m², planted in 2009 | Diameter at ground height | 1, 2, 3, 4 | ACPS, FASY, LAEU, PSME, QUPE |
| FORBIO - Zedelgem | Belgium | www.treedivbelgium.  ugent.be | 42 plots in 1 site with a density of 781 trees per 1764 m² plot, planted in 2009/2010 | Diameter at ground height | 1, 2, 3, 4 | BESP, FASY, PISY, QURO, TICO |
| ORPHEE | France | www.facebook.com/  orpheeexperiment | 256 plots in 1 site with a density of 100 trees per 400 m² plot, planted in 2008 | Tree height | 1, 2, 3, 4, 5 | BESP, QUPY, QURO, QUIL, PIPI |

**Table S1.3.** Characteristics of forest exploratories

| **Forest name** | **Country** | **Forest type** | **Number of plots** | **Species richness** | **Target tree species** |
| --- | --- | --- | --- | --- | --- |
| North Karelia | Finland | Boreal forest | 28 | 1, 2, 3 | PIAB, PISY ,BESP |
| Białowieża Primeval Forest | Poland | Hemiboreal forests, nemoral coniferous and mixed broadleaved-coniferous forests | 43 | 1, 2, 3, 4, 5 | BESP , CABE, PIAB, PISY, QUSP |
| Hainich National Park | Germany | Beech forest | 38 | 1, 2, 3, 4, 5 | FASY, FREX, QUSP, PIAB, ACPS |
| Carpathian mountains | Romania | Carpathian beech forest type (temperate deciduous) | 28 | 1, 2, 3, 4 | PIAB, ABAL, FASY, ACPS |
| Central southern Tuscany | Italy | Thermophilous deciduous forest | 36 | 1, 2, 3, 4, 5 | QUCE, QUIL, CASA, QUSP, OSCA |
| Alto Tajo Natural Park | Spain | Mediterranean mixed forest | 36 | 1, 2, 3, 4 | PISY, PINI, QUFA, QUIL |

**Table S2.** All tree species that occurred in any of the inventory, experimental or exploratory data sets.

| *ABAL - Abies alba Mill.* | *ABCO - Abies concolor (Gordon) Lindl. ex Hildebr.* | *ABGR - Abies grandis* |
| --- | --- | --- |
| *ACCA - Acer campestre L.* | *ACMO - Acer monspessulanum L.* | *ACOP - Acer opalus Mill.* |
| *ACPL - Acer platanoides L.* | *ACPS - Acer pseudoplatanus L.* | *AEHI - Aesculus hippocastanum L.* |
| *ALGL - Alnus glutinosa (L.) Gaertn.* | *ALIN - Alnus incana (L.) Moench* | *ARUN - Arbutus unedo L.* |
| *BESP - Betula spec. -* combined *B. pendula* and *B. pubescens* | *BUSE - Buxus sempervirens L.* | *CABE - Carpinus betulus L.* |
| *CASA - Castanea sativa Mill.* | *CEAT - Cedrus atlantica (Endl.) Carrière* | *CESI - Cercis siliquastrum L.* |
| *COAV - Corylus avellana L.* | *CRMO - Crataegus monogyna Jacq.* | *EUCA - Eucalyptus camaldulensis Dehnh.* |
| *FASY - Fagus sylvatica L.* | *FICA - Ficus carica L.* | *FRAN - Fraxinus angustifolia Vahl* |
| *FREX - Fraxinus excelsior L.* | *ILAQ - Ilex aquifolium L.* | *JUCO - Juniperus communis L.* |
| *JUOX - Juniperus oxycedrus L.* | *JUPH - Juniperus phoenicea L.* | *JUTH - Juniperus thurifera L.* |
| *LADE - Larix decidua Mill.* | *LAEU - Larix x eurolepis* | *LAKA - Larix kaempferi (Lamb.) Carrière sec. Franco* |
| *LASI - Larix sibirica* | *MASY - Malus sylvestris Mill.* | *OLEU - Olea europaea L.* |
| *OSCA - Ostrya carpinifolia* | *PHLA - Phillyrea latifolia L.* | *PIAB - Picea abies (L.) H.Karst.* |
| *PICA - Pinus canariensis Sweet ex Spreng.* | *PICE - Pinus cembra L.* | *PIHA - Pinus halepensis Mill.* |
| *PINI - Pinus nigra J.F.Arnold* | *PIPI2 - Pinus pinea L.* | *PIPI - Pinus pinaster* |
| *PIRA - Pinus radiata D.Don* | *PISI - Picea sitchensis (Bong.) Carrière* | *PISY - Pinus sylvestris L.* |
| *PIUN - Pinus uncinata Mill. ex Mirb.* | *PITE - Pistacia terebinthus L.* | *PLOC - Platanus occidentalis L.* |
| *POP - Populus spp.* | *PONI - Populus nigra L.* | *POTR - Populus tremula L.* |
| *PRU - Prunus spp.* | *PRAV - Prunus avium L.* | *PRDO - Prunus domesticaL.* |
| *PRMA - Prunus mahaleb L.* | *PRPA - Prunus padus L.* | *PSME - Pseudotsuga menziesii (Mirb.) Franco* |
| *PYCO - Pyrus communis L.* | *QUE - Quercus spp.* | *QUCA - Quercus canariensis Willd.* |
| *QUCE - Quercus cerris* | *QUFA - Quercus faginea Lam.* | *QUIL - Quercus ilex L.* |
| *QUPY - Quercus pyrenaica Willd.* | *QURO - Quercus robur L.* | *QURU - Quercus rubra L.* |
| *QUSP - Quercus spec. - combines Q. petraea and Q. pubescens Willd. (Q. Humilis)* | *QUSU - Quercus suber L.* | *ROPS - Robinia pseudacacia L.* |
| *SAAL - Salix alba L.* | *SAAT - Salix atrocinerea Brot.* | *SACA - Salix caprea L.* |
| *SACI - Salix cinerea L.* | *SANI - Sambucus nigra L.* | *SOAR - Sorbus aria (L.) Crantz* |
| *SOAU - Sorbus aucuparia L.* | *SODO - Sorbus domestica L.* | *SOTO - Sorbus torminalis (L.) Crantz* |
| *TABA - Taxus baccata L.* | *TICO - Tilia cordata* | *TIL - Tilia spp.* |
| *TIPL - Tilia platyphyllos Scop.* | *ULGL - Ulmus glabra Huds.* | *ULMI - Ulmus minor Mill.* |

**Fig. S3.** Climate conditions across the three research approaches (extracted from the WorldClim dataset (Hijmans *et al.* 2005)). Histograms of the forest inventory plots are shown in the background in yellow. Tree diversity experiments are marked with star symbols and forest exploratories are marked with circles.


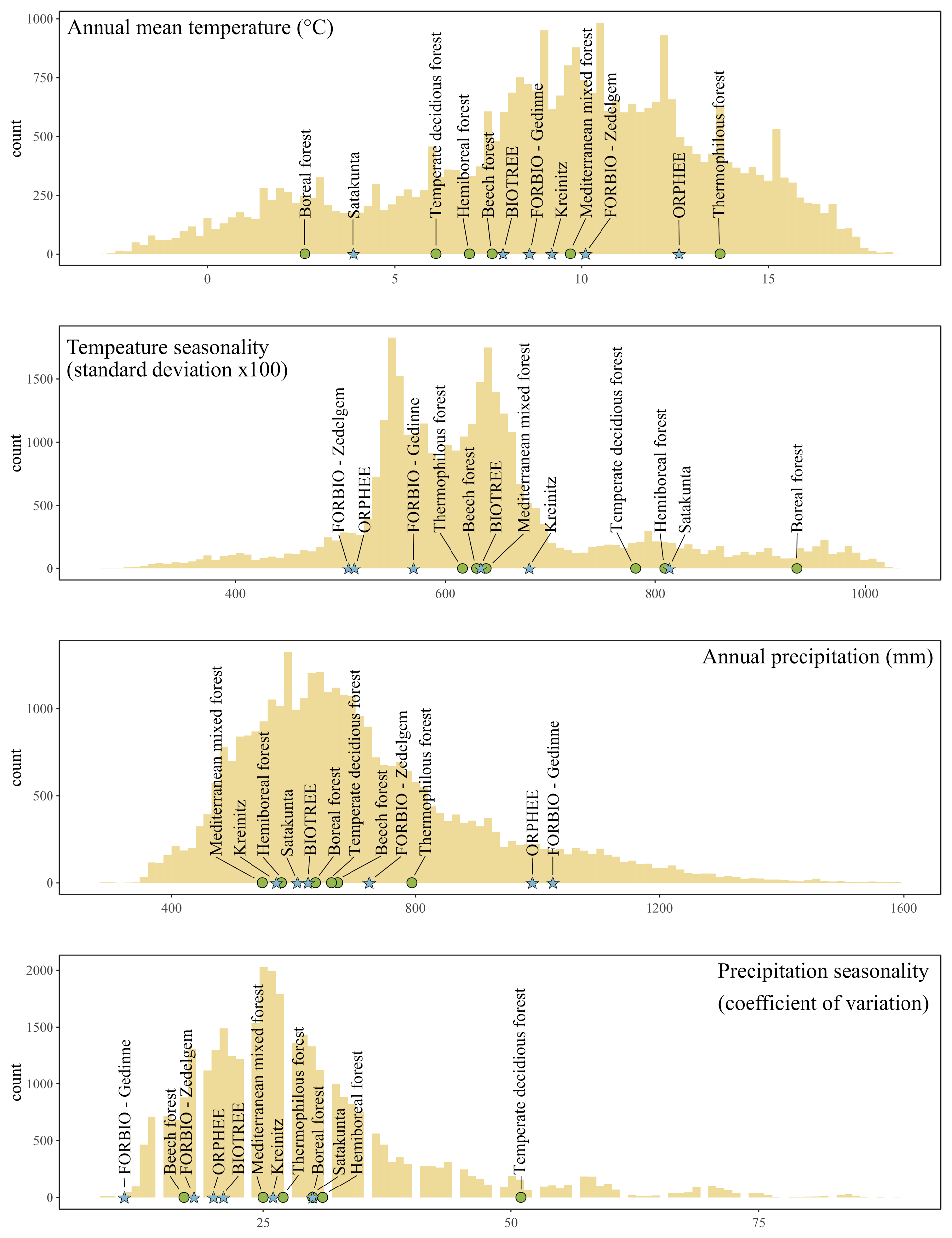


**Table S4.** Species compositions that are shared between the three different research approaches (Inv - inventory approach, Exper - experimental approach, Explor - exploratory approach). Colouring indicates if the respective tree species composition is represented in the two compared approaches.

| **Species composition** | **NFIs / Exper** | **NFIs / Explor** | **Exper / Explor** | **Species composition** | **NFIs / Exper** | **NFIs / Explor** | **Exper / Explor** | **Species composition** | **NFIs / Exper** | **NFIs / Explor** | **Exper / Explor** |
| --- | --- | --- | --- | --- | --- | --- | --- | --- | --- | --- | --- |
| ABAL | no | yes | no | BESP QURO | yes | yes | yes | PIAB PSME | yes | no | no |
| ABAL ACPS FASY | no | yes | no | CABE | no | yes | no | PIAB PSME QURO | yes | no | no |
| ABAL ACPS FASY PIAB | no | yes | no | CABE PIAB | no | yes | no | PIAB QURO | yes | yes | yes |
| ABAL ACPS PIAB | no | yes | no | CABE PIAB QURO | no | yes | no | PINI | no | yes | no |
| ABAL FASY | no | yes | no | CABE PISY | no | yes | no | PINI PISY | no | yes | no |
| ABAL FASY PIAB | no | yes | no | CABE PISY QURO | no | yes | no | PINI PISY QUFA | no | yes | no |
| ABAL PIAB | no | yes | no | CABE QURO | no | yes | no | PINI PISY QUFA QUIL | no | yes | no |
| ACPS | yes | yes | yes | CASA | no | yes | no | PINI QUFA | no | yes | no |
| ACPS FASY | yes | yes | yes | CASA QUIL | no | yes | no | PINI QUFA QUIL | no | yes | no |
| ACPS FASY FREX | no | yes | no | CASA QUIL QUSP | no | yes | no | PINI QUIL | no | yes | no |
| ACPS FASY FREX PIAB | no | yes | no | CASA QUSP | no | yes | no | PIPI2 | yes | no | no |
| ACPS FASY PIAB | no | yes | no | FASY | yes | yes | yes | PIPI2 QUIL | yes | no | no |
| ACPS FASY QUSP | no | yes | no | FASY FREX | yes | yes | yes | PIPI2 QUIL QUPY | yes | no | no |
| ACPS FREX | no | yes | no | FASY FREX PIAB | yes | yes | yes | PIPI2 QUPY | yes | no | no |
| ACPS PIAB | no | yes | no | FASY FREX QUSP | no | yes | no | PIPI2 QUPY QURO | yes | no | no |
| ACPS PSME | yes | no | no | FASY PIAB | yes | yes | yes | PIPI2 QURO | yes | no | no |
| ALGL | yes | no | no | FASY PIAB PISY | yes | no | no | PISY | yes | yes | yes |
| ALGL BESP | yes | no | no | FASY PIAB PSME | yes | no | no | PISY QUFA | no | yes | no |
| ALGL BESP PISY | yes | no | no | FASY PIAB PSME QURO | yes | no | no | PISY QURO | no | yes | no |
| ALGL PIAB | yes | no | no | FASY PIAB QURO | yes | no | no | PSME | yes | no | no |
| BESP | yes | yes | yes | FASY PIAB QUSP | no | yes | no | PSME QURO | yes | no | no |
| BESP FASY PISY | yes | no | no | FASY PISY | yes | no | no | QUFA | no | yes | no |
| BESP FASY PISY QURO | yes | no | no | FASY PISY QURO | yes | no | no | QUFA QUIL | no | yes | no |
| BESP PIAB | yes | yes | yes | FASY PSME | yes | no | no | QUIL | yes | yes | yes |
| BESP PIAB PISY | yes | yes | yes | FASY QURO | yes | no | no | QUIL QUPY | yes | no | no |
| BESP PIAB PISY QURO | no | yes | no | FASY QUSP | no | yes | no | QUIL QURO | yes | no | no |
| BESP PIAB QURO | no | yes | no | FASY TICO | yes | no | no | QUIL QUSP | no | yes | no |
| BESP PIPI2 | yes | no | no | FREX | yes | yes | yes | QUPY | yes | no | no |
| BESP PIPI2 QUPY QURO | yes | no | no | FREX PIAB | yes | yes | yes | QUPY QURO | yes | no | no |
| BESP PIPI2 QURO | yes | no | no | FREX PIAB PISY | yes | no | no | QURO | yes | yes | yes |
| BESP PISY | yes | yes | yes | FREX PISY | yes | no | no | QURO QUSP | no | yes | no |
| BESP PISY QURO | no | yes | no | PIAB | yes | yes | yes | QUSP | no | yes | no |
| BESP QUPY | yes | no | no | PIAB PISY | yes | yes | yes |  |  |  |  |
| BESP QUPY QURO | yes | no | no | PIAB PISY QURO | no | yes | no |  |  |  |  |

**Appendix S5.** Sampling design of national forest inventory plots.

Data from the national forest inventories (NFIs) of Finland, Sweden, Germany, Wallonia and Spain were compiled as part of the Inventory Platform of FunDivEUROPE. To select comparable data from the different inventories, only those trees with a diameter at breast height (dbh) of 10 cm and plots with consecutive surveys and no indication of harvest between survey dates were included in the analyses (see (Baeten *et al.* 2013; Ratcliffe *et al.* 2016) for more details).

FINLAND

The Finnish data is a subset of permanent sample plots established to follow changes in forest vegetation. Data included in this study is from two surveys: 1985 to 1986 and 1995. The sample plots are located on forest land in a systematic grid across the country (Mäkipää & Heikkinen 2003; Tomppo & Tuomainen 2009) forming a regular network of clusters. The size of the grid and the number of plots within each cluster depends on the location. In Southern Finland, the grid is 16 by 16 km square with four plots in each cluster at 400 m intervals. In Northern Finland, the grid is a 24 by 32 km rectangle with three plots per cluster at 600 m intervals.

The plot size depends on the dbh of the sample trees: 100 m² if trees < 10.5 cm in dbh, and 300 m² if trees >10.5 cm in dbh. All plots are located in intensively managed forests, in which suppressed trees were thinned.

SWEDEN

We received data from the permanent sample tracts of the Swedish NFI. The inventory uses a randomly planned regular sampling grid and includes about 4,500 permanent tracts, each surveyed every five years (Fridman *et al.* 2014). Plots in the first census were surveyed between 2003 and 2005 and plots in the second census were surveyed between 2008 and 2010. The tracts are rectangular and have different dimensions depending on the location within the country. Each tract has between 4 and 8 circular sample plots.

Trees greater than 1.3 m high are sampled in two different plot sizes depending on the dbh of the tree: 40-99 mm dbh: 3.5 m radius; and greater than 100 mm dbh: 10 m radius.

GERMANY

We received data from the first two German NFIs. The first inventory was surveyed between 1986 and 1990 (undertaken in West Germany only) and the second inventory was surveyed between 2001 and 2002. The mean survey period was 12 years.

The German NFI is based on a systematic rectangular grid, the dimensions of which are determined by the Federal State; the standard size is 4 by 4 km and it is intensified in some States to either 2.83 by 2.83 km or 2 by 2 km (Polley *et al.* 2009). In each grid square is a quadratic tract of 150 m in length. Each corner of the tract has a sample plot and the tracts are surveyed if at least one of the corners is in forest.

Trees with a minimum dbh of 10 cm, in the first inventory, and 7 cm, in the second inventory, were surveyed based on callipered angle count sampling using a basal area factor of 4 m^2^ ha^-1^.

WALLONIA

The Walloon NFI follows a systematic non-stratified sampling methodology on a 1 km by 0.5 km grid (Rondeux & Wagner 2009). One circular sampling plot is located within each grid intersection. Areas are sampled if the area of land is greater than 0.1 ha and has at least 10% covered by a forest canopy (trees must be able to reach a minimum of 5 m). Plots in the first census were surveyed between 1994 and 2003 and plots in the second census were surveyed between 2008 and 2011.

The inventory employs a variable plot size depending on the circumference of the tree: Trees with a circumference between 20-69 cm: 4.5 m radius; 70-119 cm: 9 m radius; and greater than 120 cm: 18 m radius.

SPAIN

We used data from the permanent sample plots of the second and third Spanish NFIs. Plots were surveyed between 1986 and 1996 and between 1997 and 2007, with a mean survey period of 10 years. The sample plots of the Spanish NFI are on a systematic 1 km^2^ grid in forested areas of the country, and are not grouped in tracts but simply one plot in each grid square (Villaescusa & Díaz 1998; Villanueva 2005).

The Spanish NFI used a variable radius plot size depending on the dbh of the sample trees; each plot has four nested subplots of 5, 10, 15 and 25 m radius and the minimum dbh for a tree to be recorded within a subplot is 7.5 cm, 12.5 cm, 22.5 cm and 42.4 cm, respectively.

**Fig. S6.** Locations of the mixed and monospecific plots (altogether 16,773 plots) retained in the inventory approach after the omission the mono-mix comparisons above the 10 percent percentile (see Fig. S9).

**
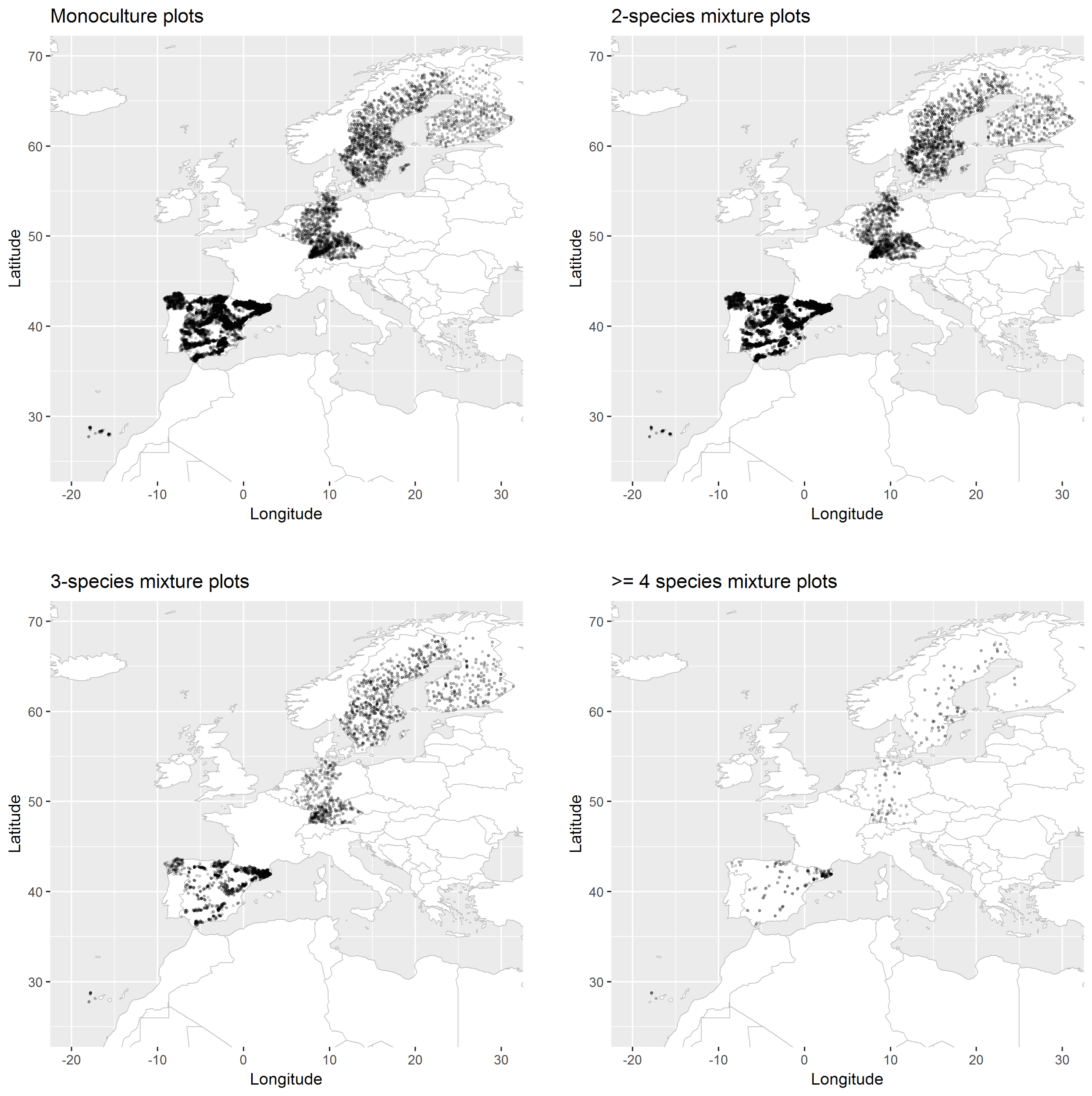
**

**Appendix S7.** Tree growth measurements in the exploratory platform.

In the exploratory platform tree growth was estimated as yearly radial stem increment (mm year^-1^) between 1990 and 2010 as measured from wood cores that were extracted at breast height between March and October of 2012. In each plot these wood cores were taken from 12 trees in monospecific and six trees per species in mixture plots (except in Poland where only five cores per species were taken in each plot). Each sample was later cross dated against an averaged reference curve obtained from all chronologies that were measured on the same species at the same site. After excluding samples with poor agreement we ended up with 2926 tree ring chronologies and omitted two plots in Italy due to lack of data. The process of radial growth measurements is described in detail in (Jucker *et al.* 2014a).

**Appendix S8.** Data preparation to compare the effect of species mixing across all research approaches.

1. SUBSET PLOTS

| **Inventories** | Retained only those plots with multiple survey dates and that were neither harvested nor managed in between subsequent surveys. |
| --- | --- |
| **Experiments** | - |
| **Exploratories** | - |

2. CALCULATE SPECIES GROWTH ESTIMATES AND PLOT CHARACTERISTICS

| **Inventories** | *Species growth estimate*: average increment in basal area ha^-1^ year^-1^ basal area (2nd survey)^-ha^ for each focal tree species  *Species proportions*: summed basal area ha^-1^ of the focal species divided by summed basal area ha^-1^ of all trees (2nd survey)  *Community density*: sum of basal area ha^-1^ of all trees (2nd survey)  *Community heterogeneity*: mean basal area of all trees divided by the respective standard deviation (2nd survey)  *Climate variables*: Mean annual temperature, temperature seasonality and annual precipitation were extracted from the World Clim dataset  *Forest type*: Based on the EEA Technical Report 9 |
| --- | --- |
| **Experiments** | *Species growth estimates*: mean diameter at ground or breast height, tree height or basal area basal area ha^-1^ per species in each plot  *Species proportions*: not accounted since species were planted in equal proportions  *Community density*: - not available  *Community heterogeneity*: - not available |
| **Exploratories** | *Species growth estimates*: average increment in basal area (S7) ha^-1^ year^-1^ basal area^-1^ for each focal tree species  *Species proportions*: summed basal area ha^-1^ of the focal species divided by summed basal area ha^-1^ of all trees  *Community density*: sum of basal area ha^-1^ of all trees  *Community heterogeneity*: mean basal area of all trees divided by the respective standard deviation  *Climate variables*: Mean annual temperature, temperature seasonality and annual precipitation were extracted from the World Clim dataset  *Forest type*: Based on the EEA Technical Report 9 |

3. DETERMINE SPECIES RICHNESS PER PLOT

| **Inventories** | Species richness levels were assigned based on species proportions:  1: One dominant species > 90%; 27,975 plots  2: Two dominant species, together > 90%; 14,478 plots  3: Three dominant species, together > 90%; 4,436 plots  Higher species richness3: > Three dominant species, together > 90%; 865 plots  Any non-focal/non-dominant species must not exceed 10%.  Plots that did not meet these criteria were filtered out. |
| --- | --- |
| **Experiments** | - |
| **Exploratories** | - |

4. MATCH SIMILAR MIXED AND MONOSPECIFIC PLOTS

| **Inventories** | We applied the following procedure to assign, for each tree species within each forest type the species growth in monospecific and mixed plots that should be as similar as possible with regard to the community density, heterogeneity and climate conditions.   - For each plot the values of community density, heterogeneity and climate conditions were standardized (divided by mean and standard deviation). - Based on these standardized values we calculated the euclidean distance between all plots in which this species occurred within the respective forest type. - We applied a nearest neighbour algorithm that assigned pairs of monospecific and mixed plots that were most similar (i.e. with the lowest Euclidean distance) while at the same time minimizing the summed Euclidean distance between all pairs (based on a binomial generalized linear model, R-package *MatchIT*, *(Ho et al. 2007, 2011)*. The histogram of all euclidean distances is shown in Fig. S9. - Assigned pairs with a distance above the 90% percentile were omitted to not compare very different mixed and monospecific plots (see Fig. S9). |
| --- | --- |
| **Experiments** | Tree species in mixed and monospecific plots are, by design, growing in very comparable conditions. |
| **Exploratories** | Tree species in mixed and monospecific plots are, by design, growing in very comparable conditions. |

5. CALCULATE GROWTH SUMMARY STATISTICS

| **Inventories** | - Within each forest type and each national forest inventory we calculated for each species: - Mean and standard deviation of species growth together with the number of plots in monospecific, two-, 3- and higher species richness mixtures. |
| --- | --- |
| **Experiments** | - Within each tree diversity experiment we calculated for each species: - Mean and standard deviation of species growth together with the number of plots in monospecific, two-, 3- and higher species richness mixtures. |
| **Exploratories** | - Within each forest type we calculated for each species: - Mean and standard deviation of species growth together with the number of plots in monospecific, two-, 3- and higher species richness mixtures. |

6. CALCULATE EFFECT SIZES

| **Inventories** | - Within each forest type we calculated for each species: - The log response ratio from the contrasting species growth in all mixed vs. all monospecific plots - These effect sizes were calculated separately for each level of species richness (two-, three- and higher species mixtures). The same set of monospecific plots was thereby contrasted with different sets of mixture plots (representing the different levels of species richness) |
| --- | --- |
| **Experiments** | - Within each experiment we calculated for each species: - The log response ratio from the contrasting species growth in all mixed vs. all monospecific plots - These effect sizes were calculated separately for each level of species richness (two-, three- and higher species mixtures). The same set of monospecific plots was thereby contrasted with different sets of mixture plots (representing the different levels of species richness) |
| **Exploratories** | - Within each forest type we calculated for each species: - The log response ratio from the contrasting species growth in all mixed vs. all monospecific plots - These effect sizes were calculated separately for each level of species richness (two-, three- and higher species mixtures). The same set of monospecific plots was thereby contrasted with different sets of mixture plots (representing the different levels of species richness) |

Log response ratios were calculated as $lnR =ln (\frac{\hat{X}{}_{mix}}{\hat{X}{}_{mono}})$

**Fig. S9.** Histogram of Euclidean distances between assigned mixed and monospecific plots in the inventory approach; calculated from standardized values of mean annual temperature, temperature seasonality, annual precipitation, precipitation seasonality, slope of the plot and the sum and coefficient of variation of the basal area of all tree individuals (m² ha^-1^). The dotted line indicates the 90% percentile above which mixed-monoculture-comparisons were omitted due to the high differences in plot conditions.


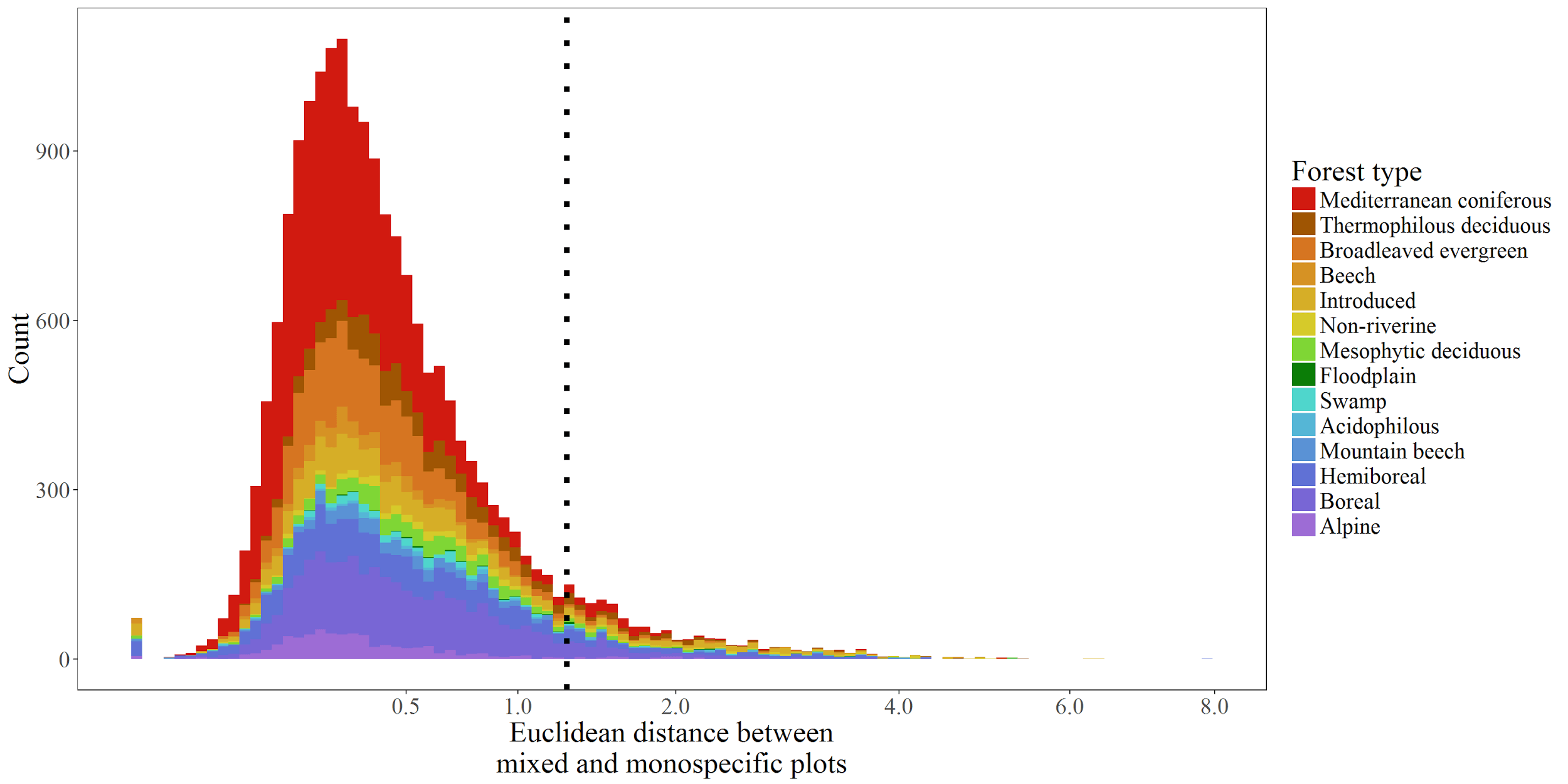


**Fig. S10.** Comparison of tree species mean effect sizes (log response ratios) of growth in mixed compared to monospecific plots obtained from three different research approaches (experimental, exploratory and inventory approach). Depicted are the mean effect sizes of only those species, species compositions and forest types that were shared between the compared research approaches (a: inventories versus experiment, b: exploratories versus experiments, c: inventories versus exploratories, d: inventories versus exploratories when species responses were separated by forest type). Abbreviations: ABAL: *Abies alba* Mill., ACPS: *Acer pseudoplatanus* L., BESP: *Betula spec*., ALGL: *Alnus glutinosa* (L.) Gaertn., CABE: *Carpinus betulus* L., CASA: *Castanea sativa* Mill., FASY: *Fagus sylvatica* L., FREX: *Fraxinus excelsior* L., PIAB: *Picea abies* (L.) H.Karst., PINI: *Pinus nigra* J.F.Arnold, PIPI2: *Pinus pinea* L., PISY: *Pinus sylvestris* L., PSME: *Pseudotsuga menziesii* (Mirb.) Franco, QUFA: *Quercus faginea* Lam., QUIL: *Quercus ilex* L., QUPY: *Quercus pyrenaica* Willd., QURO: *Quercus robur* L., QUSP: *Quercus spec* - combines *Q. petraea* and *Q. pubescens Willd. (Q. Humilis)*(Table S2).


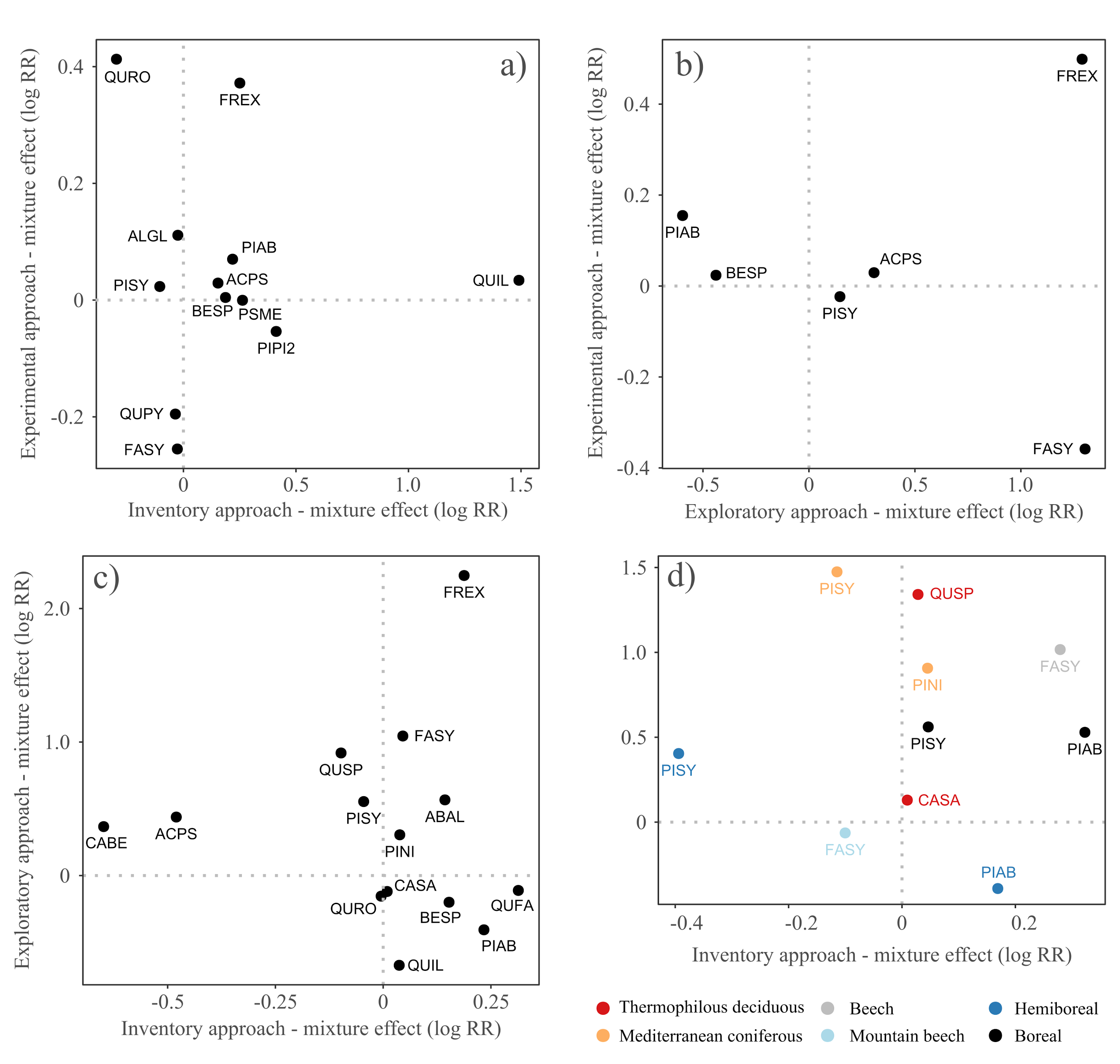

Supplement: Supplementary file 1 [file ECE3-9-11254-s001.docx]
